# Ethambutol and visual assessment in England: current practice and recommendations

**DOI:** 10.1038/s41433-023-02643-4

**Published:** 2023-06-22

**Authors:** Sally MacVinish, David McMaster, Tanya Moledina, Surinder K. Tamne, Jane Ashworth, Sarah R. Anderson

**Affiliations:** 1 UK Health Security Agency, London, UK; 2 Imperial College London, London, UK; 3 Manchester Royal Eye Hospital, Manchester, UK

**Keywords:** Respiratory tract diseases, Risk factors

## Abstract

**Background:**

Standard treatment for tuberculosis (TB) in children and adults includes an initial two-month course of ethambutol, a drug that in rare cases can cause optic neuropathy and irreversible vision loss. There is a lack of clear guidance on what vision assessments are needed before and during treatment with ethambutol, with the Royal College of Ophthalmologists, National Institute for Health and Care Excellence, British National Formulary and British Thoracic Society offering different guidance. We aimed to assess how vision is routinely tested in patients treated with ethambutol in TB services across England.

**Methods:**

An online survey developed by Public Health England was sent to all TB services in England in 2018 to assess current practice and inform the development of best practice recommendations for visual assessment of patients treated with ethambutol for TB.

**Results:**

Sixty-six TB professionals from across England responded, a response rate of 54%. The results showed variations in practice, including when to omit ethambutol from treatment, the timing and frequency of visual assessment, the type of visual assessment, referral processes and management of visual changes.

**Conclusion:**

This national survey highlights the need for clear guidelines on the testing of vision for patients taking ethambutol at recommended doses, before and during treatment. We suggest a pragmatic approach to visual assessment to reduce variation in practice, proposing a stepwise pathway for patients on standard TB treatment for local adaptation.

## Introduction

Ethambutol is one of four first-line drugs used to treat tuberculosis (TB). In 2017 the Royal College of Ophthalmologists (RCOphth) issued a statement recommending ‘a baseline visual assessment’ before starting ethambutol [1]. This followed their prior recommendation in 2010 that ‘a screening method for children seems unnecessary and impractical’ [2]. This change was prompted by two incidents of severe visual loss due to delayed diagnosis of optic neuropathy from ethambutol. Despite the risk of irreversible visual loss, there are currently no consistent UK guidelines on how vision should be assessed before or during treatment with ethambutol. The National Institute for Health and Care Excellence (NICE), British National Formulary (BNF) and British Thoracic Society (BTS) all offer different guidance (Table 1) [3–5]. NICE recommends ethambutol for 2 months as part of standard treatment for active TB in adults and children, with rifampicin and isoniazid for 6 months and pyrazinamide for 2 months. The NICE TB guidelines do not include any guidance on visual assessment for those treated with ethambutol but recommends that people with co-existing TB and eye disease should be treated by specialists experienced in managing both [3]. The BTS TB Drug Monographs recommend a baseline assessment of visual acuity and colour vision discrimination with monthly symptom screens for routine monitoring, and also recommend monthly visual acuity, colour vision discrimination and symptom screening for higher risk patients (i.e., doses >15/mg/kg/day or children or renal impairment) [5]. The BNF recommends that visual acuity is tested by Snellen chart prior to commencing ethambutol treatment, and that in young children there is routine ophthalmological monitoring [4].

**Table 1:** Summary of visual acuity, colour vision and symptom screen recommendations for patients treated with ethambutol.

	Visual acuity	Colour vision	Symptom screen	Other
NICE [3]	No recommendation	No recommendation	No recommendation	Specialist management if co-morbid TB and eye disease
BNF [4]	Baseline assessment	No recommendation	No recommendation	Ophthalmological monitoring of young children
BTS [5]	Baseline assessment & six-monthly^a^	Baseline assessment & six-monthly^a^	Monthly	-
RCOphth [1]	Baseline assessment	Baseline assessment	Baseline assessment	-

The National Institute for Health and Care Excellence (NICE), British National Formulary (BNF), British Thoracic Society (BTS), Royal College of Ophthalmologists (RCOphth).

^a^monthly for high risk patients ( > 15 mg/kg/day; children; renal impairment).

The incidence of visual impairment in patients treated with standard doses of ethambutol is estimated at 1–2%, with the risk of optic neuropathy increasing with factors such as smoking, renal disease and dose of ethambutol [1, 6, 7]. The American Academy of Ophthalmology National Registry of Drug-Induced Ocular Side Effects report a dose related incidence of visual impairment, with 50% of patients at a dose of 60–100 mg/kg/day developing optic neuropathy compared to 1% of patients at doses less than 15 mg/kg/day [8]. The dose of ethambutol for daily dosing in the BNF is 15 mg/kg once daily for adults and 20 mg/kg once daily for children [4]. Ethambutol related optic neuropathy is likely driven by mitochondrial dysfunction due to the metal chelating effects of ethambutol and may only affect the small calibre papillomacular bundle axons, with clinical signs often not developing for months after damage [8]. The earliest clinical findings are usually painless loss of visual acuity, colour vision abnormalities and central scotomas starting from two to five months after initiation of therapy, however vision changes may occur earlier, sometimes within days [9].

With a lack of consistency in UK guidelines we aim to describe the visual assessment practices of TB services in England and make recommendations including a stepwise pathway for the visual assessment of patients treated with ethambutol for local adaptation.

## Methods

A survey of visual assessment practices in TB services was developed by Public Health England (PHE) with specialist medical, clinical nursing and ophthalmology input. The online survey, developed on the PHE Select Survey platform was sent in 2018 to the lead TB nurse in each NHS Trust with a TB service in England; this was then followed up with a reminder email one month later. The survey included 33 questions to collect data on the number of adult and paediatric patients treated with ethambutol, and included questions on ethambutol prescribing, current vision screening practices before and during standard TB treatment for different age groups, referral processes and who conducts the vision screening. No ethics approval was required.

## Results

### Survey respondents

From 122 surveys distributed, 66 TB professionals representing TB services from each of the seven TB control boards in England responded, a response rate of 54%. Of the respondents, 92% were TB nurses and 8% were medical consultants; at least two TB services in each region in England responded to the survey. Of the 66 responses, 43 (65%) were from TB units that treat both children and adults and 23 (35%) from those who only treat adults. Eight (12%) units treated over 100 adult cases of active TB in 2016, 14 (21%) treated 51–100 and 35 (55%) treated 10–50 cases. Eight (12%) units treated over 10 paediatric cases of active TB in 2016, 5 (8%) treated 5–10 cases and 53 (80%) treated fewer than 5 cases.

### Ethambutol prescribing

All respondents include ethambutol in empiric TB treatment with 13 (20%) reporting being aware of at least one case of ethambutol-related optic nerve toxicity in 2016. Forty (61%) respondents report omitting ethambutol in patients with pre-existing visual impairment, 16 (24%) for fully sensitive TB, 19 (29%) for renal impairment, 8 (12%) for low cognition, 6 (9%) when visual assessment was not available and 2 (3%) if there was a language barrier. Of the 43 services that treat children, 15 (35%) omit ethambutol if the patient was unable to report visual disturbances due to young age.

### Visual assessment practice

**Fig. 1:**
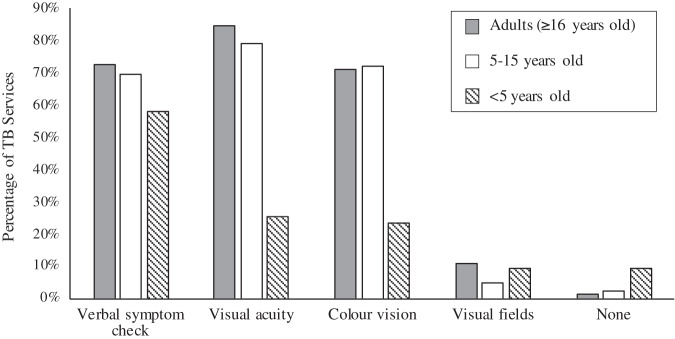
Reported baseline visual assessment of adults and children treated with ethambutol for tuberculosis.

**Fig. 2:**
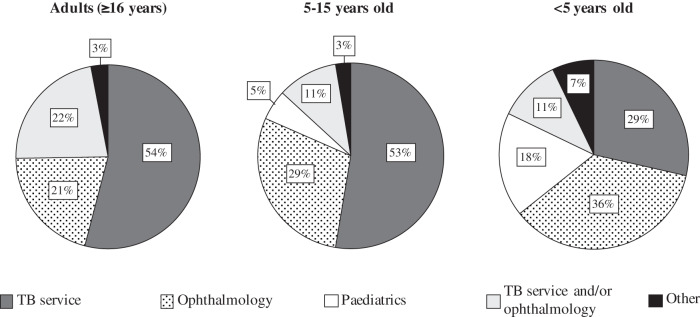
Services providing visual assessment for outpatient children and adults treated with ethambutol for tuberculosis.

Our survey showed that 60 (91%) of TB services assessed adults’ vision before starting them on ethambutol. Of those that treat children, 37 (86%) conduct baseline assessments in children 5–15 years old, and 19 (44%) conduct baseline assessments in children <5 years old. The types of visual checks undertaken at baseline varied by patient age group (Fig. 1). During treatment, visual checks vary by age group and treatment length. Overall, a symptom check was the most reported, with much smaller proportions conducting specific visual assessments (Table 2). For adult patients in the first two months of ethambutol treatment, 70% of TB Services assess vision only if patients report symptoms, 18% assess vision monthly, 12% use an alternative frequency of visual assessment, from checking at baseline to more regular testing, and 17% do no assessment. The pattern of testing is similar for paediatric patients. For patients on prolonged ethambutol treatment, verbal symptom checks are mostly conducted monthly, followed by three-monthly. Vision tests (e.g., visual acuity and colour vision) are mostly conducted in those on prolonged ethambutol treatment if visual symptoms are reported, but some TB services conduct them monthly, three-monthly, six-monthly or at other frequencies. The service that performs the vision assessment varies between outpatient age groups; for adults, 54% (34/63) of TB services check patients’ vision and 21% refer to ophthalmology services, the remainder have their vision checked by a range of services (Fig. 2). Services that treat children included referral to paediatrics (total number of responses was 38 for services treating 5–15 year olds and 28 for services treating <5 s).

**Table 2:** Percentage of tuberculosis (TB) services reporting visual assessment during the first two months, and during prolonged ethambutol treatment.

	None	Symptom check	Visual acuity	Colour Vision	Visual Fields	Other
During first two months of treatment						
<5 years old	21% (9)	53% (23)	5% (2)	5% (2)	2% (1)	5% (2)
5–15 years old	14% (6)	74% (32)	16% (7)	23% (10)	7% (3)	2% (1)
Adults (≥16 years)	17% (11)	73% (48)	21% (14)	24% (16)	9% (6)	2% (1)
During prolonged treatment						
<5 years old	14% (6)	40% (17)	14% (6)	7% (3)	7% (3)	5% (2)
5–15 years old	12% (5)	60% (26)	35% (15)	28% (12)	9% (4)	7% (3)
Adults (≥16 years)	6% (4)	77% (51)	50% (33)	42% (28)	15% (10)	6% (4)

Of the respondents, 48 (73%) of TB Services responded to a question about the training of TB nurses who conduct visual assessments. Of these respondents, 71% report on the job training in visual screening by other TB nurses, 17% report training by the ophthalmology service, 8% report a mixture of both, and 4% report either informal training or experience from a previous role. Rapid referral to ophthalmology is in place for 48 (73%) adult TB services but only 17 (26%) report a local protocol for assessing vision before and during ethambutol treatment. All respondents advised patients that if they notice any visual change after starting ethambutol they should contact their TB case manager immediately, in addition 36 (55%) also advised patients to stop their ethambutol. All respondents reported that this advice is provided verbally, with 46 (70%) also providing written information.

## Discussion

This national survey of visual assessment during ethambutol treatment shows clear variation in practice across England. These variations include when to omit ethambutol from treatment, timing and frequency of visual assessment, type of visual assessment, referral processes and management of visual changes. Ethambutol can lead to irreversible loss of vision, and these inconsistencies may risk delayed or missed diagnosis of optic neuropathy. In patients treated with ethambutol, clinical signs may not develop for months after damage and fundoscopy may be normal in the early stages. Although we did not assess the use of optical coherence tomography (OCT), it is important to note that subclinical changes in retinal nerve fibre layer (RNFL) or retinal ganglion cell layer thickness assessed via OCT may be seen before the onset of visual symptoms [10]. One study of 31 patients treated with standard dose ethambutol showed that whilst there were no change in colour vision, visual fields or on fundoscopy, retinal nerve fibre layer optical coherence tomography (RNFL-OCT) showed average RNFL thickness increased after five months [11]. In a prospective study of 50 patients treated with ethambutol for 6 months, 46% of eyes experienced subclinical damage in the form of increased visual evoked response latency, and decrease in RNFL and ganglion cell-inner plexiform layer (GCIPL) thickness from their baseline, despite no signs of clinical toxicity [12]. Changes to RNFL and GCIPL thickness has been shown to correlate with loss of visual acuity up to one year after ethambutol discontinuation [13–15]. In the United States, it is recommended that all patients on ethambutol receive regular screening by an ophthalmologist, including testing with OCT and visual evoked potentials (VEPs), and if signs of optic neuropathy are detected, ethambutol should be reduced or discontinued [9, 16].

There is a dearth of published guidelines and evidence on routine screening and optimal screening method in patients initiated on ethambutol. A review of the literature showed wide variation in screening method used to report visual outcomes with most studies involving small patient cohorts and reporting outcomes based on a range of examination and investigation findings (e.g. visual acuity, colour vision, visual fields OCT, VEPs etc.) [10, 11, 13, 14, 17, 18]. While our survey reviewed clinical assessments conducted by TB services and did not cover specialist ophthalmological investigations, this evidence emphasises the importance of ophthalmological review with specialist investigations that may detect subclinical changes. It is also important to note, in some patients visual symptoms, or reduction in visual acuity, may precede subclinical changes such as OCT-visible retinal atrophy and should not be erroneously dismissed with patients permitted to continue ethambutol. Patients with ethambutol optic neuropathy are at significant risk of progressive vision loss with OCT changes only apparent months later, by which time irreversible damage is done.

Our results show that all TB services surveyed in England use ethambutol to treat TB. The most common reason for omitting ethambutol was pre-existing visual impairment, as per current RCOphth, BNF and BTS guidance [1, 4, 5]. However, there are avoidable service factors for omitting ethambutol, including lack of ability to assess vision and language barriers. Timing, method and who performs the visual assessment varies across England. Of the respondents, 29% of TB services report not having an ophthalmology rapid referral system in place, with 74% without a local protocol for assessing vision before and during ethambutol treatment. These avoidable service factors increase the risk of delayed diagnosis of ethambutol induced optic neuropathy. This study also shows that only 17% of TB nurses have formal visual assessment training from the ophthalmology service, with the vast majority receiving in-house training from fellow TB nurses. There is currently no clear guidance on which service should train TB nurses. It is likely that a lack of consistent guidance underpins the reported variations in practice. This highlights the need to develop staff training materials and appropriate patient information, in addition to clear, unified guidelines for visual assessment of patients treated with ethambutol to reduce variation in practice, which in turn should improve patient outcomes.

Limitations of the study include the response rate, and the risk that there may be different practices in the areas which did not respond. This is more likely to be because of a lack of services or local guidance rather than more robust pathways and so the recommendation reflects the minimum standard which should be provided. TB lead nurses were surveyed as they manage most routine patient care, and although TB nurses may not prescribe ethambutol, they would be aware of all patients on anti-tuberculosis medication. This study highlights the need for further research into the optimal pathways for visual assessment of patients treated with ethambutol, including with ophthalmologists, specialist neuro-ophthalmologists, paediatric infectious disease specialists and TB clinical teams with the aim to generate expert consensus guidelines.

### Recommendations

**Fig. 3:**
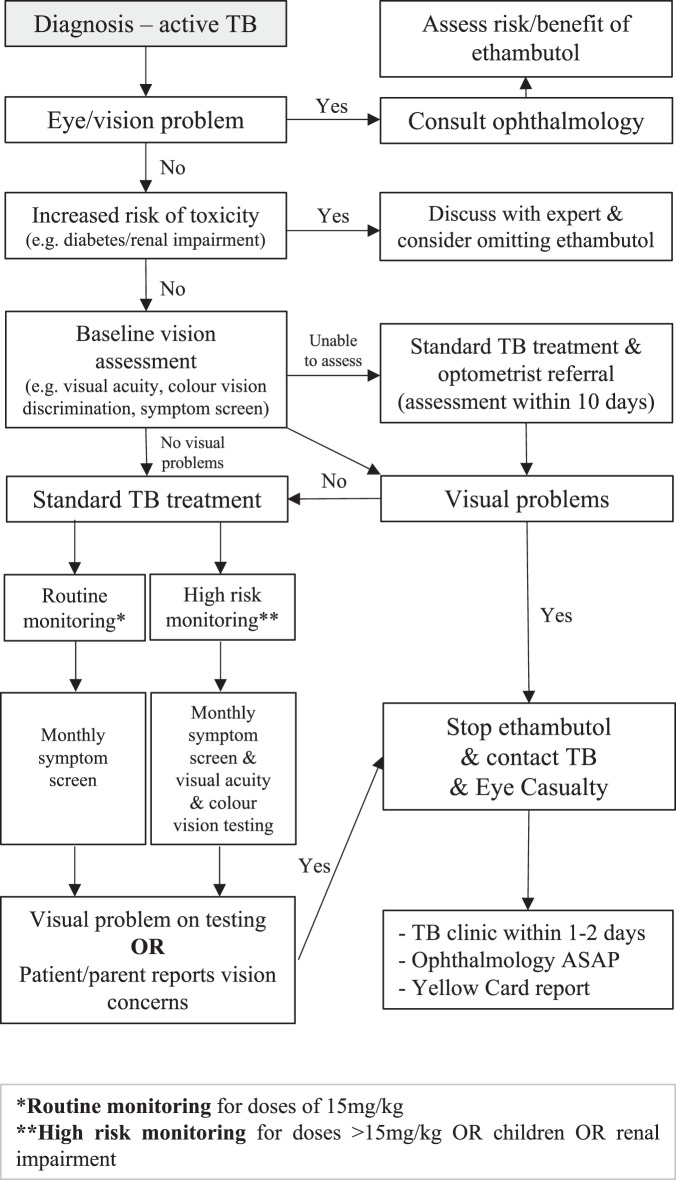
Vision testing for active tuberculosis (TB) flow chart.

Ethambutol related optic neuropathy is rare, and evidence for best practice in screening methods is limited. However, given the potential for irreversible vision loss a pragmatic approach to visual assessment is required. We propose a stepwise pathway for local adaptation (Fig. 3). At diagnosis of TB a clinical history should be taken including any risk factors for vision loss (e.g., pre-existing eye disease or impaired vision). If found, advice should be sought from ophthalmology before starting treatment with ethambutol. If there are comorbidities increasing the risk of ethambutol toxicity (e.g., renal impairment or diabetes) there may be dose adjustment or monitoring required and advice should be sought from experts (including ophthalmology), otherwise ethambutol may need to be omitted if treating fully sensitive TB.

If there are no risk factors for ethambutol toxicity, we propose following the ethambutol guidance in the British Thoracic Society TB Drugs Monograph with minor adaptations [5]. This recommends all patients have a baseline assessment including verbal symptom screen, visual acuity, and colour vision testing, followed by monthly symptom screens. For those with no risk factors on prolonged treatment, six-monthly visual acuity and colour vision discrimination are recommended. In adults, the TB manager could undertake these assessments, and for children, if cooperative, a paediatrician. If the patient is not cooperative for baseline visual assessment in clinic they should be reviewed by an optometrist for assessment. During treatment, patients and parents of children should be advised to immediately report any concerns about vision and should be counselled that optic neuropathy can occur at any dose despite regular ophthalmic exams, and that the vision loss can be severe and irreversible. If visual problems develop, ethambutol should be stopped, and the patient promptly assessed by an ophthalmologist by attending an Eye Casualty.

For patients at higher risk of toxicity, children or those with renal impairment, monthly vision assessments including symptom screens, visual acuity and colour vision testing should be performed. These recommendations should identify visual changes in a timely manner and be practicable for most TB Services. If a patient’s vision cannot be assessed in clinic, they should be referred to an optometrist and/or orthoptist for assessment, and we recommend within 10 days. This assessment should include symptom screen, visual acuity and colour vision. Detecting early subclinical visual changes (e.g., via OCT) is beyond the scope of TB services and should be considered by optometry and ophthalmology services locally. TB services should consider monitoring and evaluating these changes to practice for impact on patient outcomes.

We propose that TB services establish rapid referral pathways with local optometry and ophthalmology services for those patients at higher risk of optic neuropathy, or when changes are detected on visual testing. This should lead to a rapid ophthalmology review to minimise treatment disruption and reduce the risk of visual loss. It is reasonable to expect that all TB service staff conducting visual assessments should receive formal visual assessment training to ensure safety and quality.

## Conclusion

This national survey highlights the need for clear UK guidelines on the testing of vision for patients treated with ethambutol, before and during treatment. Ethambutol can lead to irreversible loss of vision, and the variations in practice shown by this survey may result in a delayed or missed diagnosis of optic neuropathy. Whilst ocular toxicity in adults and children is rare, we recommend that TB services assess patients’ vision according to their risk factors for vision loss and ethambutol toxicity. This approach is largely based on the BTS Drug Monograph advice for ethambutol, with adaptations including recommendations for referral to ophthalmology and optometrist services as needed. For those on prolonged courses of ethambutol, clinical monitoring is even more important. These recommendations suggested and the stepwise pathway proposed should help decrease variation in practice and improve patient outcomes.

## Summary

### What was known before

- Ethambutol use can lead to irreversible visual loss. Despite this, there is no consistent guideline on what vision assessments are needed before and during treatment.

### What this study adds

- An analysis of variation in practice between tuberculosis services in England on timing, frequency, and types of visual assessment for patients treated with ethambutol. We suggest a pragmatic approach to visual assessment to reduce variation in practice, and propose a stepwise pathway for local adaptation.

## Data Availability

Data available upon request.
